# Surgical Repair of Luxation of the Superficial Digital Flexor Tendon in Dogs Using a Calcaneal Chondroplastic-like Technique—Three Cases

**DOI:** 10.3390/ani13091468

**Published:** 2023-04-26

**Authors:** Riccardo Botto, Sara Sassaroli, Luca Pennasilico, Angela Palumbo Piccionello

**Affiliations:** 1 School of Biosciences and Veterinary Medicine, University of Camerino, Via Circonvallazione 93/95, 62024 Matelica, MC, Italy; riccardo.botto@unicam.it (R.B.); luca.pennasilico@unicam.it (L.P.); angela.palumbo@unicam.it (A.P.P.); 2 Clinica Veterinaria Monleale, Corso Roma 8/a, 15059 Monleale, AL, Italy

**Keywords:** superficial digital flexor, luxation, calcaneal chondroplastic-like technique, dog

## Abstract

**Simple Summary:**

Luxation of the superficial digital flexor (SDF) tendon is an infrequent orthopedic condition in dogs. The rupture of the medial or lateral retinacular insertion of the SDF on the calcaneal tuberosity allows the tendon to luxate laterally or medially, respectively. Obesity, microtrauma, torsional forces, and morphological abnormality of the calcaneus are predisposing factors and, in particular, a shallow surface to the calcaneal tuberosity as a consequence of underdeveloped medial or lateral processes. To date, surgical treatments have not focused on the correction of any detected anatomical defect of the shape or size of the calcaneal tip but on repairing and reinforcing the damaged retinaculum. The purpose of this report is to describe the technique and the clinical outcome of dogs with SDF tendon luxation treated using a calcaneal chondroplastic-like technique. This surgery helped to accommodate and stabilize the SDF tendon in the calcaneal groove. This treatment showed excellent short- and long-term outcomes, without signs of re-luxation and lameness during follow-ups. These preliminary data show that calcaneal chondroplasty associated with retinaculum repair might be a valid option for the treatment of luxation of the SDF tendon. Further studies are required to determine the real success of the surgical technique proposed in this report.

**Abstract:**

The purpose of this report is to describe the technique and the clinical outcome of three dogs affected by superficial digital flexor (SDF) tendon luxation treated using a calcaneal chondroplastic-like technique. A German Pinscher with bilateral and lateral SDF tendon luxation, a Griffon Nivernais with medial SDF tendon luxation following self-mutilation of the IV toe, and an American Staffordshire Terrier with a lateral luxation and having undergone calcaneal chondroplasty and primary repair of the retinacular tissues. A fibrocartilage flap covering the calcaneal groove was elevated, the subchondral bone was removed from beneath it, and the flap was pressed back into the deepened sulcus, keeping its distal attachment as a hinge point. The SDF tendon was reduced, and its tracking along the deepened groove was ensured. Furthermore, the torn retinacular attachment was repaired. Clinical follow-ups at 4 and 8 weeks and 1 year apart showed no signs of lameness and no SDF tendon re-luxation. The calcaneal chondroplastic-like technique led to a satisfactory outcome with no complications. This technique is relatively straightforward, requires no implants, and is also successful without postoperative immobilization of the tarsal joint. Further cases are required to determine its benefits and its risks compared to conventional surgery.

## 1. Introduction

Luxation of the superficial digital flexor (SDF) tendon is an infrequent orthopedic condition in dogs [1,2,3,4,5,6]. The incidence of luxation of the SDF tendon is higher in Shetland sheepdogs and in Collies, but it is also seen in other breeds [1,3,4,5,7,8].

The SDF muscle arises with gastrocnemius muscle mainly from the lateral supracondylar tuberosity of the femur and from the lateral sesamoid. The superficial digital flexor muscle runs deep to the lateral head of the gastrocnemius muscle and winds medially and caudally around the tendon of the gastrocnemius at the middle of the tibia. Distally, the SDF tendon forms a cap-like structure over the calcaneal tuberosity, where it inserts collaterally, melting with the medial and lateral retinaculum. At the calcaneal tuberosity, the SDF tendon glides in a groove bordered by medial and lateral processes and covered by fibrocartilage. A synovial bursa, which extends proximally and distally from the tuber, is present underneath the tendon, separating it from the gastrocnemius tendon and the calcaneus. The tendon divides into four parts and continues distally to its final insertion on the middle phalanges of the second to fifth digits [9].

Rupture of the medial or lateral retinacular insertion of this tendon on the calcaneal tuberosity allows the tendon to luxate laterally or medially, respectively [1,2,3,4,5,6,7,10], but lateral luxations of the SDF tendon occur more commonly than medial luxations because the lateral insertion is more prominent and distinct than the insertion on the medial aspect of the calcaneus [1,2,3,4,5,7,10]. Obesity, repeated microtrauma, torsional forces, and skeletal malformations, such as flattened calcaneal tuberosity [4,6,7,11,12], are predisposing factors. SDF tendon luxation seems to be associated with vigorous activity and may be attributed to a rotational force acting on the point of insertion at the tuber [3,10,13].

The degree of lameness ranges from mild to severe depending on whether the luxation is acute or delayed and may be intermittent because dogs may be lame only when the tendon is luxated [3,4,10,14]. During the episode of intermittent tendon luxation, a popping sensation may be appreciated during the flexion and extension of the hock, and accurate palpation of the tendon allows a diagnosis. Moderate swelling on either side of the calcaneus may be detected as a result of accompanying bursitis [10,14].

The treatment of choice to restore normal function is to surgically repair the damaged soft tissue and place the SDF tendon in the center of the groove. Surgical management involves interrupted non-absorbable sutures placed from the edge of the tendon to the retinacular insertion [10,14]. A recent study has shown that the use of non-absorbable sutures is associated with having a more successful outcome compared to absorbable material [13]. Redundant retinacular tissue may result from stretching and can be imbricated or excised [10].

In the literature, postoperative immobilization of the tarsus is recommended for a minimum of 4 weeks to avoid surgical treatment failure [2,4,5,6,7,14]. A rigid external coaptation seems to be correlated with good outcomes compared to non-rigid immobilization [13], but common are soft tissue complications due to the external bandage of the distal limb [13,15]. Recently, some authors have proposed the use of a temporary restraining pin to protect the primary repair in order to avoid postoperative immobilization of the tarsal joint and its possible complications. Taking into account the retrospective nature of the study and the low number of cases, the authors have stressed the usefulness of a temporary restraining pin in the surgical repair of SDF tendon luxation [16].

Over the years, little importance has been given to the dysplasia of the calcaneal tuberosity despite it being proposed as a predisposing factor [4,6,10]. Surgical treatments have focused on repair, reinforcement, and protection of the retaining tissue during healing and not on the correction of any underlying anatomical defect to the shape of the calcaneal groove. Recent reports have reported that if the groove of the calcaneus is intraoperatively observed to be shallow or absent, with a distolateral slant, it can be useful to perform block recession calcaneoplasty of the calcaneal [17] or tuber abrasion calcaneoplasty [18] in order to deepen the groove of the calcaneal tuberosity, analogously to the surgical treatment of the patellar luxation. A calcaneal chondroplastic-like technique could also be useful in these subjects who do not show significant structural changes in calcaneal tuberosity, as it would allow us to keep the tendon along the groove waiting for the healing of the repaired soft tissues. All this would allow us to avoid immobilization of the operated tarsus for a long time and reduce the risk of postoperative re-luxation.

The purpose of this case series report was to evaluate the short- and long-term outcomes of four chondroplastic-like calcanea to surgical repair luxations of the SDF tendon without postoperative immobilization.

## 2. Case Report

**Figure 1:**
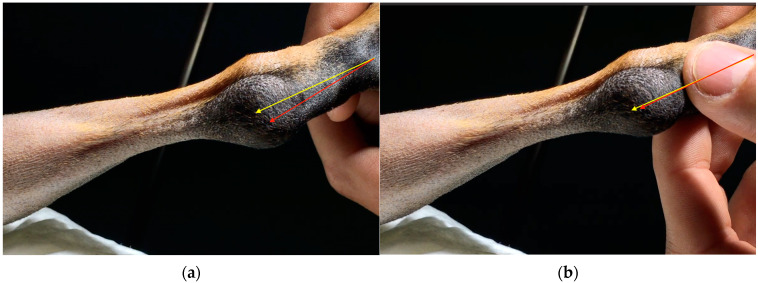
(**a**) Lateral luxation of SDF tendon and (**b**) SDF tendon located at the center of calcaneal tuberosity. The red arrow represents the tendon; the yellow arrow represents the position that the tendon should have along the anatomical axis of the hind limb.

*Case 1.* A 10-month-old dog, female, indoor German Pinscher weighing 7 kg with a body condition score (BCS) of 4 (scale from 0 to 9), was presented as a referral because of skipping lameness of the left hind limb, which had started 2 months earlier following a jump from the couch. The lameness was not responsive to NSAIDs. The orthopedic examination revealed a moderate lameness (grade II on a scale from 0 to IV) [19] of the left hind limb and swelling at the left calcaneal tuberosity. Moderate pain was revealed on the digital palpation of the calcaneal tuberosity. Flexion of the left tibial-tarsal joint caused lateral luxation of the SDF tendon (Figure 1a). During the extension of the joint, the tendon returned to its correct position (Figure 1b). No other orthopedic abnormalities were found either in the affected limb or in the contralateral one. Dorsoplantar and mediolateral radiographic projections of the left tibial-tarsal joint highlighted the radiopacity of pericalcaneal soft tissue without osteoarticular changes.

Surgical repair, as described below, was carried out, and a modified Robert Jones splint was applied for 24 h after the surgery. Meloxicam (0.1 mg/kg) once a day and amoxicillin + clavulanic acid (12.5 mg/kg) twice a day was prescribed for 7 days, and only short walks on the leash were to be gradually increased during the two months postoperative rehabilitation was recommended.

Four and 8 weeks after surgery, the dog had no lameness (grade 0) [19], the tendon appeared stable during flexion-extension movements of the hock, and the swelling at the site of the retrocalcaneal bursa disappeared.

**Figure 2:**
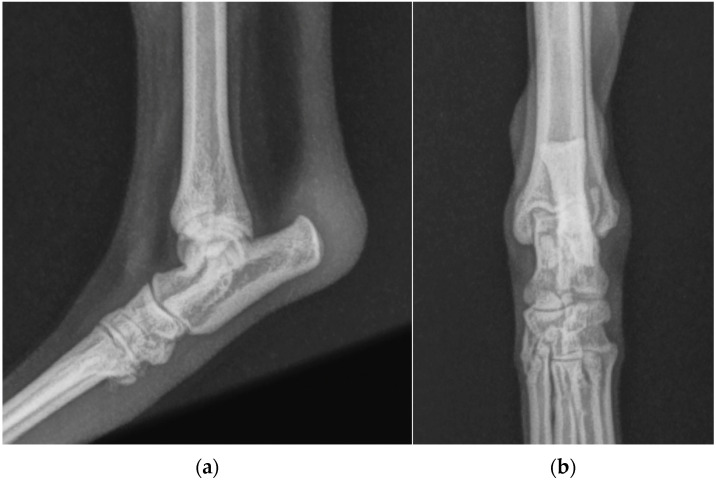
(**a**) Mediolateral and (**b**) dorsoplantar radiographic projections of the right tibial-tarsal joint’s highlighted radiopacity of pericalcaneal soft tissue without osteoarticular changes.

**Figure 3:**
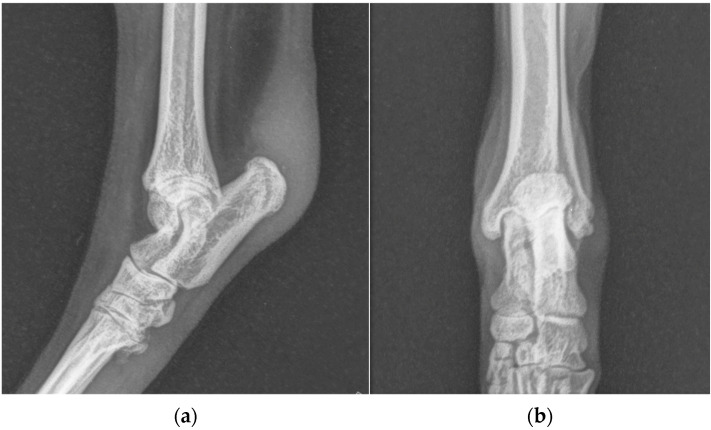
(**a**) Mediolateral and (**b**) dorsoplantar radiographic projections of the right tibial-tarsal joint 12 months after the surgery. The images highlighted radiopacity of pericalcaneal soft tissue and rearrangement of bone tissue at the tip of calcaneal tuberosity without secondary osteoarthrosis.

One years after surgery, the patient was presented with the same clinical evidence in the right hind limb (grade II) [19]. SDF tendon lateral luxation of the right hind limb was diagnosed following a clinical and radiographic examination similar to the previous one (Figure 2). The SDF tendon of the left hind limb was perfectly in place, and dorsoplantar and mediolateral radiographic images of the tibial-tarsal joint did not detect mild radiopacity of pericalcaneal soft tissue and rearrangement of bone tissue at the tip of the calcaneal tuberosity without secondary osteoarthrosis. The dog underwent surgery and postoperative treatment as described previously. At the follow-up clinic and radiographic examination, 4 and 8 weeks and 12 months after the intervention, no complications were reported (Figure 3).

**Figure 4:**
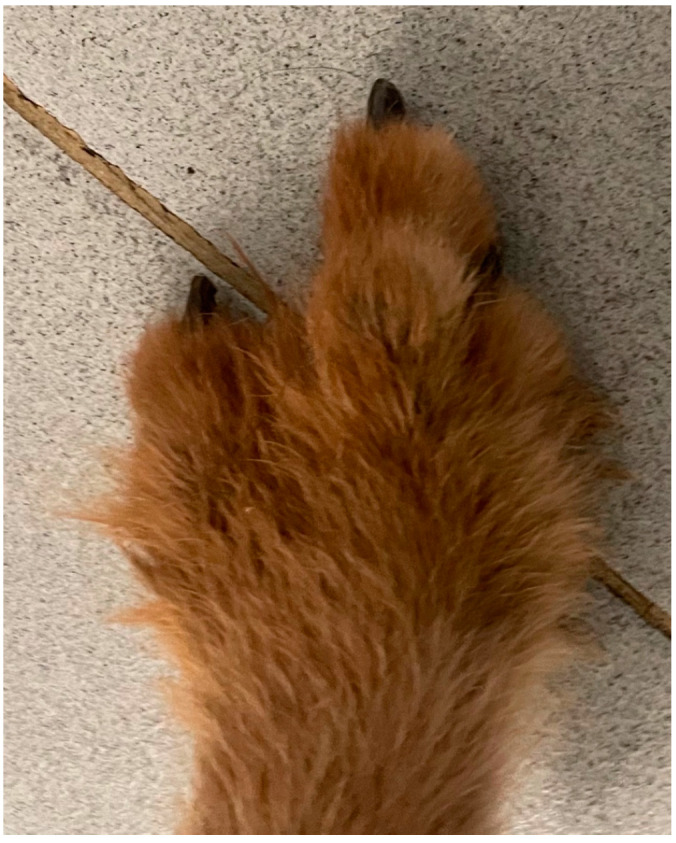
Griffon Nivernais dog’s foot without IV toe.

**Figure 5:**
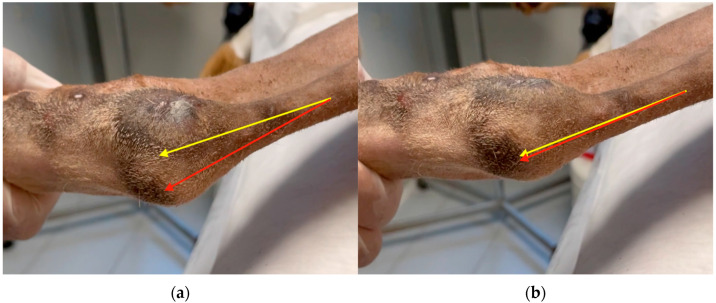
(**a**) SDF tendon (red arrow) luxates medially during flexion of the left tibial-tarsal joint. (**b**) During the extension of the joint, the tendon returned to its correct position along the anatomical axis of the hind limb (yellow arrow).

*Case 2.* A 5-year-old hunting dog, male, outdoor Griffon Nivernais weighing 38 kg, BCS 4, was presented because, intermittently, non-weight-bearing lameness of the right hind limb was reported by the owner (grade II) [19]. Fifteen months before, the patient was subjected to neurorrhaphy of the sciatic nerve following a boar injury (cranio-dorsal to the ischial tuberosity). The patient recovered after 12 months, and during that time, he ate his own IV toe (I, II, and III thick phalanges) due to a sensory deficit (Figure 4). The owner reports that during this period, the dog resumed hunting activity. An orthopedic examination revealed a soft swelling involving the proximal aspect of the calcaneal tuberosity of the right hind limb. Flexion and extension of the tibial-tarsal joint revealed medial luxation of the SDF tendon. (Figure 5) Radiographs revealed no skeletal abnormalities and surgical repair was performed. A modified Robert Jones splint was applied for 24 h. Drug therapy and physical activity restrictions were prescribed, as in case 1.

Not respecting postoperative recommendations to wear the Elizabethan collar, two weeks after surgical repair, a granuloma for licking the wound appeared at the surgical suture. It was conservatively treated. Clinical examination revealed no re-luxation of the tendon. Lameness and displacement of the SDF tendon and radiographic changes were not detected at 4 and 8 weeks and 1 year after surgery.

**Figure 6:**
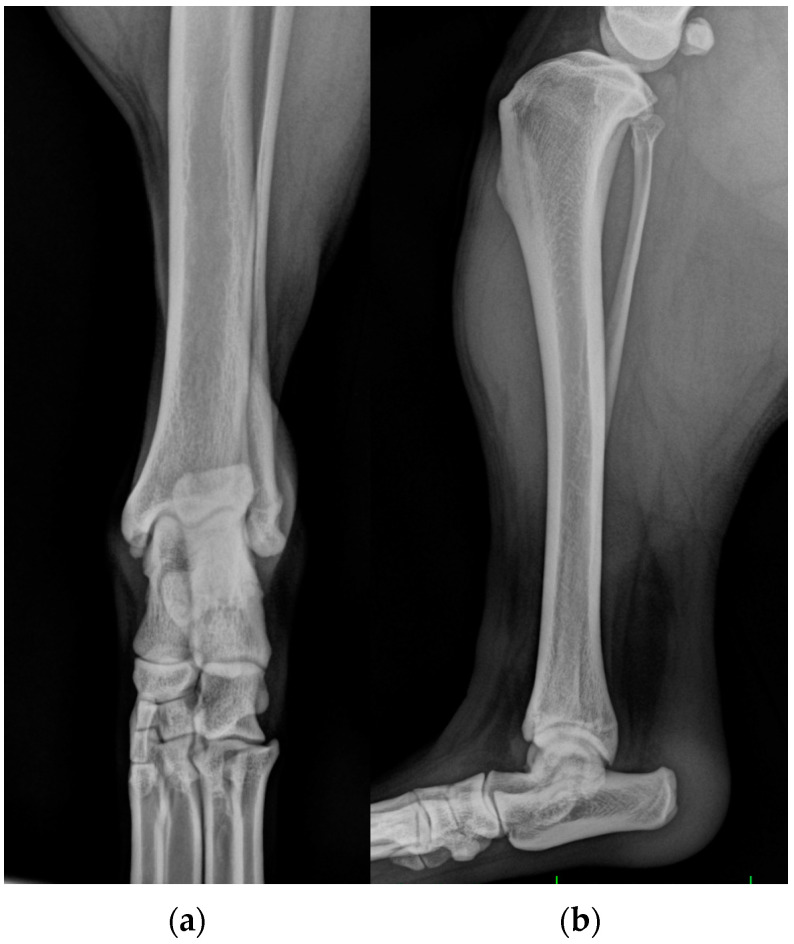
(**a**) Dorsoplantar and (**b**) mediolateral radiographic projections of the left tibial-tarsal joint showed soft swelling over the calcaneus without osteoarticular changes.

*Case 3.* A 3-year-old, spayed female American Staffordshire Terrier dog of 22 kg of body weight, BCS 5, was presented as a referral with a suspected left cranial cruciate ligament lesion (grade II) [19]. Physical and radiographic examination of stifles revealed no abnormality. Moderate swelling was revealed over the left calcaneal tuberosity, and pain was elicited upon digital palpation of the left calcaneal tendon. Lateral SDF tendon luxation was diagnosed on palpation. Dorsoplantar and mediolateral radiographic projections of the left tibial-tarsal joint showed soft swelling over the calcaneus (Figure 6).

Surgical stabilization of the SDF tendon was performed, and postoperative care was provided as previously described (cases 1 and 2). The follow-up at 4 and 8 weeks and 12 months did not show any complication or relapse.

### Surgical Technique

After methadone administration [0.2 mg/kg intramuscularly (IM)] (Senfortan), anesthesia was induced with propofol (4–8 mg/kg intravenously (IV) administered to effect) and maintained with isoflurane in oxygen. Analgesia was provided with a target-controlled infusion of fentanyl. Pre-operative cefazolin (22 mg/kg IV) was administered at anesthesia induction and then every 90 min until completion of surgery.

The patient was placed in sternal recumbency, with the pelvic limbs hanging over the edge of the surgical table. The affected limb was aseptically prepared for surgery from the metatarsal-phalangeal joints to the coxo-femoral joint.

The skin incision began on the caudolateral side of the common calcanean tendon just proximal to the calcaneal tuberosity in the case of medial SDF tendon luxation (case 2). In the case of lateral SDF tendon luxation (cases 1 and 3), the skin incision began on the caudomedial side of the common calcanean tendon just proximal to the calcaneal tuberosity. As it curved distally, it remained respectively caudolateral or caudomedial to the plantar midline of the calcaneus and ended at the middle third of the calcaneus.

The skin and the subcutaneous fascia were reflected to expose the retinaculum and the bursa calcanei. The border of the SDF tendon was visualized through the retinaculum. The retinaculum and the bursa calcanei were incised parallel to the lateral border of the SDF tendon (case 2) or parallel to the medial border of the SDF tendon (cases 1 and 3) and continued proximally to allow separation of the tendon from the gastrocnemius tendon. The SDF tendon was retracted with a Hohmann retractor to expose the fibrocartilage covering the tuberosity and the calcaneal groove.

Approximately 90° of flexion of the stifle and the tarsal joint was performed to tension the gastrocnemius muscle’s mechanism and to provide visualization and protection of the calcaneus tendon insertion. A “trochlear chondroplasty” procedure was performed on the calcaneal grooves to accommodate the SDF tendon by the calcaneal chondroplastic-like technique.

**Figure 7:**
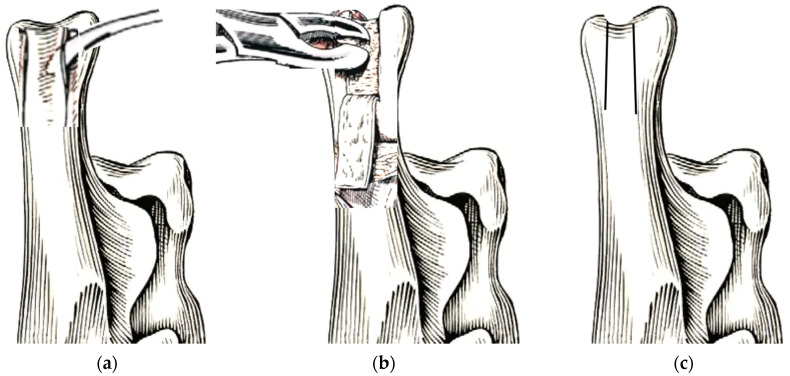
Illustrative image of calcaneal chondroplastic-like technique. (**a**) The fibrocartilage covering the calcaneal groove is incised with a scalpel blade at its proximal end, and two parallel longitudinal incisions were made along the medial and lateral processes and ended at the first third of the plantar part of the calcaneus. The fibrocartilage flap is elevated with a thin osteotome and mallet (Lambotte osteotome) from the cranio-proximal part to the caudal-distal of the calcaneus, maintaining its distal attachment. (**b**) The subchondral bone is removed with a Lambotte osteotome or a small Rongeur, and (**c**) the fibrocartilage flap is replaced into the recessed bone.

**Figure 8:**
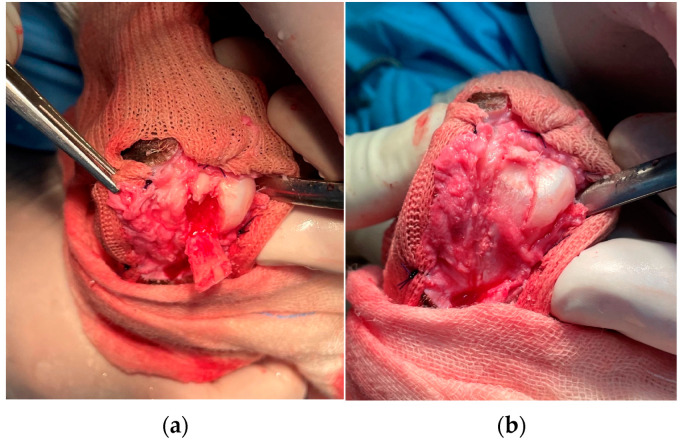
(**a**) A fibrocartilage flap covering the calcaneal groove is elevated, keeping its distal attachment as a hinge point, and the subchondral bone is removed from beneath it. (**b**) Following this, the flap is pressed back into the deepened sulcus.

The calcaneal tuberosity fibrocartilage was incised with a scalpel blade on the proximal end of the calcaneal groove, perpendicular to the medial and lateral processes. Subsequently, two parallel longitudinal incisions were made along the medial and lateral processes and ended at the first third of the plantar part of the calcaneus (Figure 7 and Figure 8). These two incisions were deepened using a manual patella luxation saw (PLX Luxation Saw C/W 15 mm Blade, Securos, USA) and a fibro-cartilage flap elevated carefully with a thin osteotome and mallet (Lambotte osteotome) from the cranio-proximal part to the caudal-distal of the calcaneus, maintaining its distal attachment. The calcaneal subchondral bone had been removed with a Lambotte osteotome or a small Rongeur in the toy breed dogs, and the pedicled fibro-cartilage flap was replaced in a recessed position, using the distal attachment as the hinge point (Figure 7 and Figure 8). The SDF tendon was reduced, and its tracking along the deepened groove was ensured in extension, flexion, and the intra–extra rotation of the tarsal joint; if tendon dislocation was noticed, a new deepening of the calcaneal groove had to be performed.

Following an abundant washing, the retinacular tissues were repaired by interrupted absorbable sutures (polydioxanone, PDS II) 2/0 in all subjects with the exception of the Pinscher (case 1), which was performed with a 3/0 suture. The subcutaneous tissues and skin were closed routinely. A modified Robert Jones splint was applied for 24 h postoperatively. The surgical time was 30 min on average.

## 3. Discussion

According to the literature in this report, 75% (three out of four reported luxations) of SDF tendon luxation are lateral. Only one in four cases (case 2) had a medial luxation, a condition considered infrequent by previous studies [1,2,3,4,5,7,10]. The medial SDF luxations are reported in combination with the concurrent longitudinal tendon tear of the SDF [6,16]. In this case, there were no tendon tears, but the tendinous insertion on the IV toe was absent. It is possible that the absence of the IV toe determined an imbalance of the forces that the SDF exerts on the plantar face of the phalanges, translating these forces on the medial portion of the foot. The increased tension on the planto-medial portion of the distal extremity of the hind limb and the resumption of physical activity of the dog may be the cause of the medial SDF tendon luxation found in this patient.

None of our patients was a Shetland sheepdog or a cross with this predisposed breed. In Shelties, an autosomal recessive defect results in a flattened calcaneal tuberosity [7], which is a predisposing factor for tendon luxation [4,6,7,11,12], but other studies also report SDF tendon luxation in racing Greyhounds [14] and in other breeds, such as Chow Chows [3], Labrador retrievers [2,13], a terrier [1], Yorkshire terriers, Samoyeds, Golden retrievers, Australian shepherd dogs [4,8], and cross-breeds [6,13]. To our knowledge, this is the first report that documents SDF tendon luxation in a German Pinscher, in an American Staffordshire Terrier dog, and in a Griffon Nivernais. It is our opinion that, in clinical practice, the disease should be considered not only in predisposed breeds but also in other breeds.

Differently from the existing knowledge, obesity does not appear to be a factor responsible for SDF luxation in the patients included in our study; all three dogs were normal-weight patients with a BCS in the normal range. Instead, according to the current literature, SDF tendon luxation is associated with vigorous activity because all patients included were hyperactive dogs [3,4,6,7,10,11,12,13]. In particular, in case 2, the manifestation of SDF tendon luxation occurred as a result of the resumption of hunting activity, later a self-injury by removing the IV toe. Until today, the partial absence of the final insertion of the SDF tendon on the middle phalanges was not reported in the literature as a predisposing occurrence.

Despite the morphological abnormality of the calcaneus proposed as a predisposing factor to SDF tendon luxation, the treatments described in the literature have not focused on the correction of any detected anatomical defect of the shape of the calcaneal tip. Repair and reinforcement of damaged retinaculum and protection of tissues during healing are the main goals to date of the authors. Recently, Jury et al. [16] suggested that a kind of sulcoplasty of calcaneal tuberosity would be logical to perform if a significant morphological abnormality was encountered intraoperatively. The latest study reported a successful long-term outcome through abrasion calcaneoplasty and retinaculum embrication in 12 dogs. A more recent case report described a block recession calcaneoplasty of the calcaneal tuber with retinaculum repair using an anchor screw and a temporary restraining pin for treating lateral SDF tendon luxation in a dog. In these studies, clear evidence of bone deformity was not observed in the radiographic images, but a flattened calcaneal groove was observed intraoperatively [17,18].

**Figure 9:**
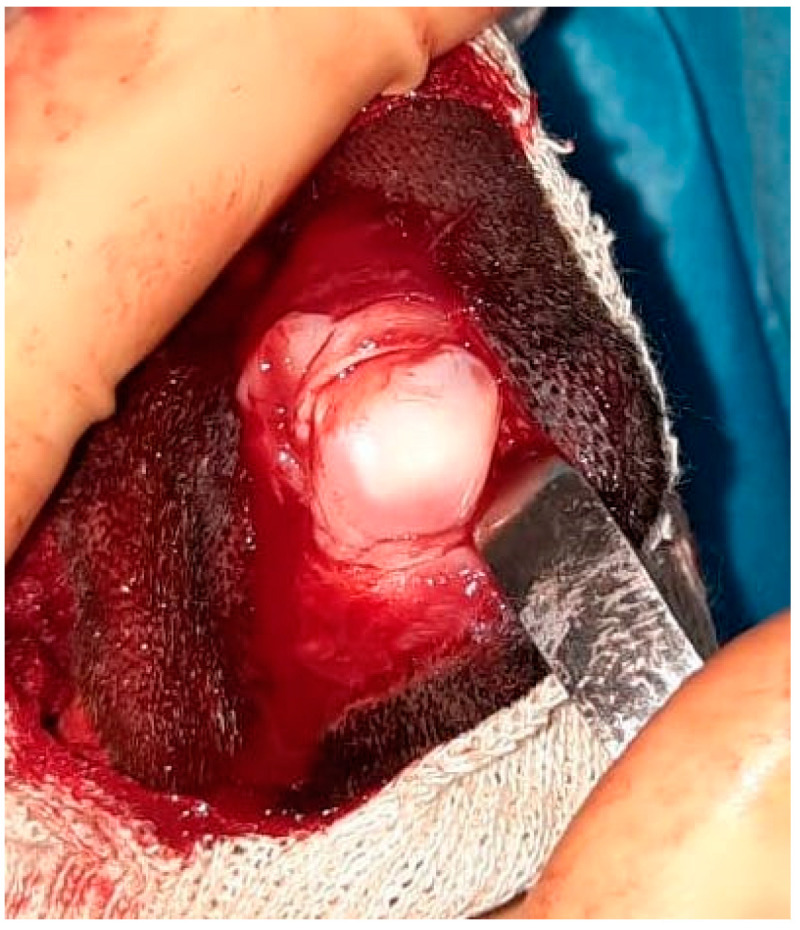
Intraoperative image of dome-shaped fibrocartilage surface of the calcaneal tuberosity of the German Pincher.

In all our cases, the radiographic projections of the tibial-tarsal joint highlighted the radiopacity of the pericalcaneal soft tissue without any morphological abnormality of the calcaneus. Only the German Pinscher showed a domed fibrocartilage surface of the calcaneal tuberosity on visual inspection during the surgery (Figure 9). In our opinion, performing a calcaneal chondroplastic-like technique elevating a flap of fibrocartilage that covers the calcaneal tip could also be useful in those patients with an apparently normal calcaneal surface because the deepening of the calcaneal groove keeps the tendon in place without overloading the repaired soft tissues.

In the literature, a period of postoperative immobilizations ranging from 4 to 8 weeks in order to avoid surgical treatment failure is recommended [2,4,5,6,7,17,18] and, recently, colleagues have observed that the limb immobilization of 6 weeks or longer did not significantly affect surgical outcomes [13]. Contrary to what is suggested by the literature, all three of our patients did not need a postoperative immobilization of the tarsal joint following SDF tendon luxation repair because the deepening of the groove ensures tendon stability. A soft bandage splint was applied for 24 h after the surgery only for the purpose of reducing postoperative edema. This minimum period of immobilization allows for minimizing the soft-tissue complications associated with prolonged use of a bandage [15], reducing the postoperative recovery time. Other authors also documented the unnecessariness of limb immobilization following the use of a temporary restraining pin, which keeps the tendon in a central position on the calcaneal tip and protects the primary repair of the retinaculum [16].

In all four cases of SDF tendon luxation, the retinacular tissues were repaired by interrupted absorbable sutures (polydioxanone), and they were not embricated or trimmed. The use of a non-absorbable monofilament suture to repair the torn retinacular attachment at the calcaneus has been recommended [2,3,4,10,14,18], but despite this, some surgeons prefer a polydioxanone suture to non-absorbable monofilament sutures [8,16]. Despite recent evidence that the risk of surgical failure was 60% higher where absorbable suture material was used compared to non-absorbable suture material [13], in our report, there were no surgery failures. This result may be due to the fact that in the study of Goh et al. [13], the imbrication of the redundant retinaculum using an interrupted suture pattern was the only surgical technique for the treatment of SDF tendon luxation, without any additional temporary restraining pin [8,16] or calcaneal sulcoplasty, as in our report.

The detection of the SDF tendon in the central position of the calcaneal groove and the absence of lameness during all clinical checks (4 and 8 weeks and 1 year postoperatively) confirmed the successful outcome of the surgery. The granuloma secondary to licking the wound noticed in the Griffon Nivernais dog is the only complication detected in our case series. This complication does not depend on the technique and surgical material used, but it depends on the owner’s noncompliance with postoperative indications.

The limitation of this case series is the small number of dogs treated. Further studies are required to determine the benefits and risks of calcaneal chondroplastic-like technique, compared to conventional surgery, with and without limb immobilization and using absorbable or not suture material.

## 4. Conclusions

To the best of our knowledge, this is the first report which describes the use of a calcaneal chondroplastic-like technique and retinaculum repair to treat SDF tendon luxation without retinaculum imbrication and postoperative immobilization of the pelvic limb. These preliminary data show that the calcaneal chondroplastic-like technique associated with primary lesion repaircmight be a valid option for the treatment of luxation of the SDF tendon. It is our opinion that in all dogs with SDF tendon luxation and predisposed factors (obesity, misalignment, and hyperactivity), not only in patients with calcaneal dysplasia, the deepening of the groove can give greater stability to the tendon and promote the healing of the retinaculum, which otherwise would be continuously stressed.

## Data Availability

The data present in this study are available within the article.
